# An Upstream Open Reading Frame Represses Translation of Chicken PPARγ Transcript Variant 1

**DOI:** 10.3389/fgene.2020.00165

**Published:** 2020-02-28

**Authors:** Yankai Chu, Jiaxin Huang, Guangwei Ma, Tingting Cui, Xiaohong Yan, Hui Li, Ning Wang

**Affiliations:** ^1^Key Laboratory of Chicken Genetics and Breeding, Ministry of Agriculture and Rural Affairs, Harbin, China; ^2^Key Laboratory of Animal Genetics, Breeding and Reproduction, Education Department of Heilongjiang Province, Harbin, China; ^3^College of Animal Science and Technology, Northeast Agricultural University, Harbin, China

**Keywords:** PPARγ, 5′-untranslated region, upstream open reading frame, translational repression, gene expression

## Abstract

Peroxisome proliferator-activated receptor γ (PPARγ) is a master regulator of adipogenesis. The *PPAR*γ gene produces various transcripts with different 5′-untranslated regions (5′ UTRs) because of alternative promoter usage and splicing. The 5′ UTR plays important roles in posttranscriptional gene regulation. However, to date, the regulatory role and underlying mechanism of 5′ UTRs in the posttranscriptional regulation of PPARγ expression remain largely unclear. In this study, we investigated the effects of 5′ UTRs on posttranscriptional regulation using reporter assays. Our results showed that the five PPARγ 5′ UTRs exerted different effects on reporter gene activity. Bioinformatics analysis showed that chicken PPARγ transcript 1 (*PPAR*γ*1*) possessed an upstream open reading frame (uORF) in its 5′ UTR. Mutation analysis showed that a mutation in the uORF led to increased *Renilla* luciferase activity and PPARγ protein expression, but decreased *Renilla* luciferase and *PPAR*γ*1* mRNA expression. mRNA stability analysis using real-time RT-PCR showed that the uORF mutation did not interfere with mRNA stability, but promoter activity analysis of the cloned 5′ UTR showed that the uORF mutation reduced promoter activity. Furthermore, *in vitro* transcription/translation assays demonstrated that the uORF mutation markedly increased the translation of *PPAR*γ*1* mRNA. Collectively, our results indicate that the uORF represses the translation of chicken *PPAR*γ*1* mRNA.

## Introduction

Peroxisome proliferator-activated receptor γ (PPARγ) is a member of the PPAR subfamily of ligand-activated transcription factors. *In vitro* and *in vivo* studies have demonstrated that PPARγ is essential for adipocyte differentiation, adipocyte survival, adipocyte function, insulin sensitivity, and lipogenesis (Lehrke and Lazar, 2005; Lefterova et al., 2014). Synthetic PPARγ agonists have been used as therapeutic agents for diabetes and insulin insensitivity (Cariou et al., 2012).

The *PPAR*γ gene is controlled by multiple alternative promoters (Aprile et al., 2014; Chandra et al., 2017). Because of alternative promoter usage and splicing, the *PPAR*γ gene can produce multiple transcript variants, resulting in expression of two PPARγ protein isoforms that differ in the N-terminal. All PPARγ transcript variants differ in their 5′-untranslated regions (5′ UTRs) (Mcclelland et al., 2014). These PPARγ 5′ UTR isoforms have distinct tissue distributions (Ahmadian et al., 2013), suggesting that the 5′ UTRs may be involved in posttranscriptional and translational regulation of the *PPAR*γ gene.

The 5′ UTRs of mRNAs exert crucial roles in posttranscriptional and translational regulation. Several *cis*-regulatory elements within the 5′ UTRs have been identified, such as the 5′ cap structure (Mitchell et al., 2010), upstream open reading frames (uORFs) (Hood et al., 2009; Barbosa et al., 2013), internal ribosome entry sites (IRES) (Xia and Holcik, 2009), terminal oligo-pyrimidine tracts, secondary structures, and G-quadruplexes (Yamashita et al., 2008; Bugaut and Balasubramanian, 2012). These *cis*-regulatory elements can function via various mechanisms, controlling mRNA stability (Nasif et al., 2018), nuclear export, localization, and translation efficiency (Araujo et al., 2012). Of these *cis*-regulatory elements, uORFs have been widely studied. Bioinformatics analysis showed that about 50% of human transcripts contain uORFs (Suzuki et al., 2000; Iacono et al., 2005; Calvo et al., 2009), and experimental studies have revealed that a number of uORFs can affect the expression of the main downstream ORFs by inducing mRNA decay or by regulating translation (Iacono et al., 2005; Crowe et al., 2006; Sathirapongsasuti et al., 2011).

Given the importance of PPARγ in various physiological and pathological processes, *PPAR*γ gene regulation has been extensively studied at the genomic and transcriptional levels in recent decades (Lee and Ge, 2014). The half-life of PPARγ mRNA and protein is short and PPARγ protein can be post-translationally modified in various ways (van Beekum et al., 2009; Katsura et al., 2014), suggesting that posttranscriptional regulation is crucial for its function. However, to date, posttranscriptional regulation by the 5′ UTR has been mostly unexplored. In the present study, we investigated the posttranscriptional regulation of chicken PPARγ by the 5′ UTR. Of interest, we demonstrated that translation of chicken PPARγ transcript variant 1 (*PPAR*γ*1*) is repressed by a uORF that is absent in human and mouse PPARγ transcripts.

## Materials and Methods

### Cell Culture

Chicken embryo fibroblast (DF1) cell line was purchased from the Institute of Biochemistry and Cell Biology, Chinese Academy of Sciences, and the immortalized chicken preadipocyte cell line 1 (ICP1) was generated in our laboratory (Wang et al., 2017). All cells were grown in Dulbecco’s modified Eagle’s medium supplemented with 10% fetal bovine serum and 1% penicillin/streptomycin, at 37°C and 5% CO_2_. The culture medium was changed two to three times per week and cells were passaged 1:3 or 1:5 as needed.

### Plasmid Construction

For PPARγ 5′ UTR reporter constructs, the reporter vector psi-CHECK2 (Invitrogen, Carlsbad, CA, United States) was mutated using the Site-directed Gene Mutagenesis Kit (Beyotime Institute of Biotechnology, Shanghai, China) according to the manufacturer’s instructions, in which the initiation start codon ATG of *Renilla* luciferase was mutated to TTG, and the resultant vector was named psi-CHECK2-Mut. Then, the DNA sequences corresponding to the five different PPARγ 5′ UTRs plus initiation codon ATG were synthesized and inserted into the *Nhe*I restriction site upstream of the *Renilla* luciferase gene to create the five chicken PPARγ 5′ UTR reporter constructs: PPARγ1-5′UTR, PPARγ2-5′UTR, PPARγ3-5′UTR, PPARγ4-5′UTR, and PPARγ5-5′UTR, respectively. In these five 5′ UTR reporter constructs, the *Renilla* luciferase expression was driven by SV40 early enhancer/promoter, and these PPARγ 5′ UTRs were expressed as the respective 5′ UTRs of *Renilla* luciferase mRNAs.

To test the promoter activity of the DNA sequences corresponding to the PPARγ1 wild-type and uORF-mutant 5′ UTRs, which the uAUG was mutated to a stop codon UAG (AUG > UAG), the DNA sequences were synthesized and subcloned into the *Bam*HI and *Xho*I restriction sites of pGL3-basic vector and named pGL3-PPARγ-WT and pGL3-PPARγ-Mut, respectively. Site-directed mutagenesis of the uORF was performed by DNA synthesis (GENEWIZ, Suzhou, China).

For PPARγ expression constructs, the full-length coding sequence of PPARγ1 was PCR amplified from the cDNA derived from DF1 cells with a set of primers (forward primer: 5′-GAATTCATGGTTGACACAGAAATGCCGT-3′ and reverse primer: 5′-CCTCGAGGAGGATAAGAACTACTATCGCC-3′) and cloned into the *Bam*HI and *Eco*RI restriction sites of the pcDNA3.1 expression vector. The synthesized DNA fragments corresponding to the wild-type and uORF mutated 5′ UTR (AUG > UAG) of PPARγ1 were inserted with *Bam*HI and *Nhe*I restriction sites upstream of PPARγ ORF in pcDNA3.1 vector. The resultant vectors were named pcDNA3.1-PPARγ-WT and pcDNA3.1-PPARγ-Mut, respectively. All constructs were confirmed by DNA sequencing and restriction enzyme digestion.

### Quantitative Real-Time PCR Assays (qRT-PCRs)

Total RNA was isolated from ICP1 or DF1 cells using the RNeasy Plus Mini Kit (Qiagen, Hilden, Germany) according to the manufacturer’s protocol, and the first-strand cDNA was synthesized from 1 μg of total RNA with oligo dT or random primers using ImProm-II reverse transcriptase (Promega, Madison, WI, United States). The qPCR reactions were performed in a 20 μL reaction mixture using FastStart Universal SYBR Green Master [Rox] (Roche, Madison, WI, United States). The primers were as follows: *Renilla* luciferase (*Rluc*) (forward 5′-TGATCGAGTCCTGGGACGA-3′, reverse 5′-ACAATCTGGACGACGTCGGG-3′); wild-type and uORF-mutated PPARγ1 (forward 5′-GGAGTTTATCCCACCAGAAG-3′, reverse 5′-AATCAACAGTGGTAAATGGC-3′); *NONO* (forward 5′-AGAAGCAGCAGCAAGAAC-3′, reverse 5′-TCCTCCATCCTCCTCAGT-3′). qPCR was carried out in an ABI 7500 real-time PCR system (Applied Biosystems, Foster City, CA, United States), and PCR results were recorded as threshold cycle numbers (Ct). The fold change in the target gene expression, normalized to the expression of an internal control gene (*NONO*) and relative to the expression at time point 0 (Normann et al., 2016), was calculated using the 2^–ΔΔCt^ method (Livak and Schmittgen, 2001). The results are presented as the mean ± SEM of three independent experiments.

### Protein Isolation and Western Blot Analysis

The ICP1 cells were transfected with either pcDNA3.1-PPARγ-WT or pcDNA-PPARγ-Mut vector. At 48 h post-transfection, cells were washed twice with PBS and lysed using radioimmunoprecipitation assay (RIPA) buffer (Beyotime Institute of Biotechnology, Shanghai, China) supplemented with 1% protease inhibitor mixture. Equal amounts of protein extracts were separated by sodium dodecyl sulfate-PAGE, transferred onto Immun−Blot PVDF membranes (Millipore, Billerica, MA, United States). The membrane was blocked for 1 to 2 h at room temperature with Tris-buffered saline containing 0.1% Tween and 5% non-fat dry milk, and immunoblotted with rabbit polyclonal antibody to chicken PPARγ (1:1000 dilution) or β-actin (1:1000 dilution, ZSGB-BIO, Beijing, China) at room temperature for 1 h. Horseradish peroxidase-conjugated goat anti-mouse or goat anti-rabbit IgG (Promega, Madison, WI, United States; 1:10,000) was incubated for 1 h at room temperature and then washed four times with PBS-Tween for 20 min. The immunoreactive bands were visualized using an ECL Plus detection kit (HaiGene Biotechnology, Harbin, China). Immunoreactive protein levels were determined semi-quantitatively by densitometric analysis using the UVP system Labworks TM software 3.0 (UVP, Upland, CA, United States). Each western blot analysis was performed at least three times.

### Dual-Luciferase Reporter Assays

Transient transfections were performed using Lipofectamine 2000 (Invitrogen, Carlsbad, CA, United States). Cells were plated at 1.0 to 1.5 × 10^5^ cells per well in 24-well plates. For the 5′ UTR reporter gene assay, 1 μg of the indicated reporter constructs was transfected into each well. For the promoter reporter gene assay, 0.8 μg of the indicated reporter constructs and 0.4 μg of pRL-TK (Invitrogen, Carlsbad, CA, United States), as an internal control of transfection efficiency, were co-transfected into each well. Luciferase activity was analyzed at 48 h post-transfection, using a dual-luciferase reporter kit (Promega, Madison, WI, United States) as per the manufacturer’s instructions. All luciferase reporter assays were performed at least three times in quadruplicates.

### *In vitro* Transcription and Translation

Plasmids pcDNA3.1-PPARγ-WT and pcDNA3.1-PPARγ-Mut were linearized, purified by agarose gel electrophoresis, eluted with diethylpyrocarbonate-treated H_2_O, and quantified. Equal amounts (1 μg) of linearized DNA were used as templates for *in vitro* transcription in the T7 RiboMAX Large Scale RNA Production System (Promega, Madison, WI, United States) according to the manufacturer’s protocol. Capped mRNAs were generated using the Ribo m^7^G Cap Analog (Promega, Madison, WI, United States). The capped mRNAs were digested with DNase I and purified with the RNeasy kit (Qiagen, Hilden, Germany) and quantified. The size and integrity of the purified mRNAs were assessed by gel electrophoresis. The mRNA outputs of pcDNA3.1-PPARγ-WT and pcDNA3.1-PPARγ-Mut were analyzed by absolute qRT-PCR. *In vitro* translation reactions were performed in nuclease-treated Rabbit Reticulocyte Lysate (Promega, Madison, WI, United States) as described by the manufacturer. Equal amounts of the capped mRNA (2 μg) derived from pcDNA3.1-PPARγ-WT or pcDNA3.1-PPARγ-Mut construct were used as the template for *in vitro* translation, which was performed for 60 min at 30°C, and the reactions were stopped by transferring the tubes to ice. Biotinylated lysine residues were added to the translation reaction as a precharged ε-labeled biotinylated lysine-tRNA complex (Transcend tRNA; Promega, Madison, WI, United States) and incorporated into nascent proteins during translation. The translated protein was analyzed using a Transcend Non-Radioactive Translation Detection System (Promega, Madison, WI, United States).

### RNA Stability Assay

The stability of luciferase mRNA transcripts from the indicated constructs (PPARγ1-5′UTR-WT and PPARγ1-5′UTR-Mut) was determined by measuring the amount of *Rluc* luciferase mRNA at selected intervals: 0 (control), 3, 6, 9, and 12 h, following the addition of 5 mg/mL actinomycin D (Sigma-Aldrich, St. Louis, MO, United States) at 48 h post-transfection. Time-course intervals were chosen based on the manufacturer’s data of *luc2* mRNA half-life (approximately 2 h). For mRNA expression analysis, total RNA (1 μg) was reverse-transcribed into cDNA using the PrimeScript RT reagent Kit with gDNA Eraser (Takara, Shiga, Japan), and relative mRNA expression was determined by real-time PCR using FastStart Universal SYBR Green Master [Rox] (Roche, Madison, WI, United States) with *Rluc* primers as described above. Relative mRNA levels were normalized to the *NONO* gene and to expression at time point 0 (Normann et al., 2016) and calculated using the 2^–ΔΔCt^ method (Livak and Schmittgen, 2001).

### Bioinformatics Analysis

Online software programs used to predict the potential *cis*-regulatory elements of PPARγ 5′ UTR: StarORF^1^ (Ceraj et al., 2009), UTRscan^2^ (Grillo et al., 2010), and Reg RNA2.0^3^ (Chang et al., 2013). Preliminary RNA secondary structures were predicted using Vienna RNAfold 2.0^4^ (Hofacker, 2003). Intrinsic protein disorder analyses were made using PSIPRED protein sequence analysis workbench^5^ (Buchan et al., 2013). All bioinformatic computations were performed using default prediction parameters.

### Statistical Analysis

Experimental data were analyzed using GraphPad Prism software (GraphPad Inc., San Diego, CA, United States). The results were presented as mean ± SEM. For comparison of two groups, statistical analysis was performed using two-tailed Student’s *t*-test and linear regression. *P* values < 0.05 (^∗^) were considered significant, *P* values < 0.01 (^∗∗^) were considered highly significant. For multiple comparisons, one-way analysis of variance (ANOVA) was used to determine significance, followed by Tukey’s *post hoc* test.

### Data Availability Statement

Strains, plasmids and cell lines are available upon request. The authors affirm that all data necessary for confirming the conclusions of the article are present within the article, figures, and Supplementary Material.

## Results

### The Effects of PPARγ 5′ UTRs on Reporter Gene Expression

We previously identified five different chicken PPARγ transcript variants (*PPAR*γ 1 to 5) by 5′ rapid amplification of cDNA ends (5′ RACE) in chicken abdominal adipose tissue (Duan et al., 2015). These chicken PPARγ transcript variants encode two protein isoforms (PPARγ1 and PPARγ2) that differ in their N-terminal extension. Chicken PPARγ2 contains six additional amino acids at the N-terminus compared with PPARγ1. These five chicken PPARγ transcript variants differed in 5′ UTR sequence and length, and had different tissue distribution patterns (Duan et al., 2015), suggesting that 5′ UTRs may play a role in the posttranscriptional regulation of *PPAR*γ gene expression. To investigate the posttranscriptional regulatory roles of the five 5′ UTR isoforms on chicken *PPAR*γ gene expression, we constructed their respective 5′ UTR reporter constructs. Firstly, the ATG start codon of *Renilla* luciferase gene in the psi-CHECK2 vector was mutated to TTG by site-directed mutagenesis, and the resultant vector was named psi-CHECK2-Mut. Then, the five DNA fragments corresponding to the five different PPARγ 5′ UTRs plus the start codon ATG were synthesized and inserted at the *Nhe*I restriction site upstream of the *Renilla* luciferase gene in psi-CHECK2-Mut to yield the five chicken PPARγ 5′ UTR reporter constructs.

**FIGURE 1:**
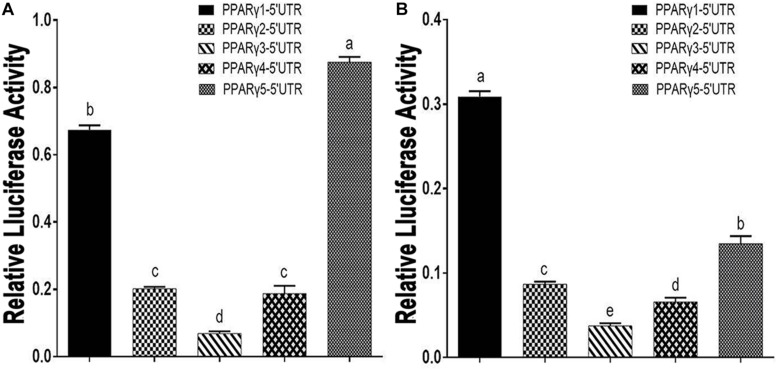
Effects of PPARγ 5′ UTR isoforms on reporter gene activity. **(A)** The luciferase activity of each of the PPARγ 5′ UTR reporter constructs was measured in DF1 cells. **(B)** The luciferase activity of each of the PPARγ 5′ UTR reporter constructs was measured in ICP1 cells. Data are expressed as mean ± SEM (*n* ≥ 3 independent experiments). Lower case letters above bars indicate the order of expression levels, “a” represents the highest expression level, “e” represents the lowest expression level. Bars with different superscripts are mutually statistically different. ANOVA followed by Tukey’s multiple comparison test was used to determine significance.

We transfected these five 5′ UTR reporters into an ICP1 cell line and a chicken embryo fibroblast (DF1) cell line and measured *Renilla* luciferase activity. The reporter gene assay showed that these five 5′ UTR reporters displayed different luciferase activities. As shown in Figures 1A,B, PPARγ1-5′UTR exhibited the highest luciferase activity in ICP1 cells and the second-highest activity in DF1 cells. PPARγ5-5′UTR exhibited the highest activity in DF1 cells and the second-highest activity in ICP1 cells. PPARγ3-5′UTR exhibited the lowest activity in both ICP1 and DF1 cells. PPARγ2-5′UTR and PPARγ4-5′UTR exhibited similar reporter activity in both ICP1 and DF1 cells. These results support our speculation that the 5′ UTR regulates chicken *PPAR*γ gene expression.

### Bioinformatics Analysis of Chicken PPARγ 5′ UTRs

**FIGURE 2:**
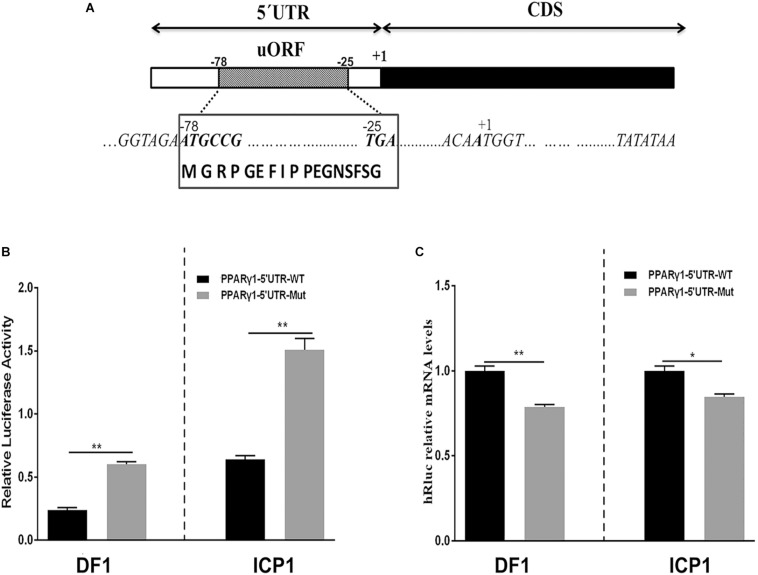
Schematic representation of PPARγ1 5′ UTR and effects of the PPARγ1 uORF mutation on *Rluc* luciferase activity and mRNA expression. **(A)** A schematic diagram of the 117-nucleotide-long PPARγ1 5′ UTR, the uORF is from nucleotides –78 to –25 of the 5′ UTR, and indicated by a striped rectangle. All positions are numbered relative to the initiation codon AUG of PPARγ transcript 1, where A is +1. The uORF encodes a 17-amino acid peptide with the amino acid sequence shown in the bottom. **(B)** The effect of uORF mutation on the luciferase reporter gene activity. The wild-type (PPARγ1-5′UTR-WT) and uORF mutant (PPARγ1-5′UTR-Mut) PPARγ1 5′ UTR reporter constructs were transfected into ICP1 and DF1 cells, respectively, and reporter gene activity was measured. Compared with the wild-type PPARγ1 5′ UTR reporter, the luciferase activity of PPARγ1-5′UTR-Mut was significantly higher than that of PPARγ1-5′UTR-WT in both ICP1 and DF1 cells (*n* ≥ 3, **P < 0.01, Student’s *t*-test). **(C)** The *Rluc* mRNA quantification by real-time RT-PCR in the ICP1 and DF1 cells transfected with the indicated reporter constructs. The relative *Rluc* mRNA levels are normalized to the expression levels of the cells transfected with the reporter PPARγ1-5′UTR-WT. Data were expressed as the mean ± SEM, *NONO* was used as the internal mRNA control. *n* ≥ 3, *P < 0.05; **P < 0.01, Student’s *t*-test.

To gain insight into the molecular mechanisms by which the 5′ UTRs regulate gene expression, we performed a bioinformatics analysis of these five 5′ UTR sequences using the online software programs StarORF, UTRscan, and RegRNA 2.0. Bioinformatics analysis showed that PPARγ1 5′ UTR contains a 54-nucleotide (nt)-long uORF (PPARγ1 uORF), PPARγ3 5′ UTR has a 12-nt-long uORF (PPARγ3 uORF) and a putative IRES element, and PPARγ5 5′ UTR has two uORFs, which are 15 and 51 nt long, respectively. No putative *cis*-regulatory elements were predicted in PPARγ2 and PPARγ4 5′ UTR sequences. The PPARγ1 uORF is located in its 5′ UTR from nucleotides −78 to −25 (relative to the start codon AUG of the PPARγ protein-coding ORF, where A is +1; Figure 2A), and the uORF AUG (uAUG) resides in a favorable Kozak consensus context, suggesting that there is a high probability that scanning ribosomes consistently initiate the translation at this uAUG codon to encode a 17-amino acid peptide (MGRPGEFIPPEGNSFSG; Figure 2A).

### The Effect of PPARγ1 uORF on Gene Expression

Upstream ORFs have emerged as a major posttranscriptional regulatory element in eukaryotic species (Wen et al., 2009). The above bioinformatics analysis showed that, of these five PPARγ 5′UTR isoforms, three contained uORFs, which led us to speculate that these uORFs may be implicated in posttranscriptional regulation of chicken PPARγ. Herein, we focused our attention on the PPARγ1 uORF. Of these five chicken PPARγ transcript variants, PPARγ transcript variant 1 (PPARγ1) is highly expressed in various chicken tissues, including abdominal adipose, spleen, and liver (Duan et al., 2015), which is consistent with our results showing that PPARγ1 5′ UTR had high reporter activity (Figures 1A,B). Unlike the other two uORF-containing 5′ UTR isoforms, PPARγ1 5′ UTR presented the largest uORF, and its uAUG was in a favorable Kozak consensus context.

To test our speculation, we investigated the effect of PPARγ1 uORF on posttranscriptional regulation of the *Renilla* luciferase reporter gene. We generated a uORF-mutated reporter construct, named PPARγ1-uORF-Mut, by mutating the uAUG of the PPARγ1 uORF to a stop codon UAG (AUG > UAG) by site-directed mutagenesis. Transient transfection and reporter gene assays showed that the luciferase activities of the mutant reporter construct (PPARγ1-uORF-Mut) were 3- and 2.5-fold higher, respectively, than those of the wild-type PPARγ1 5′ UTR reporter construct (PPARγ1-5′UTR-WT) in ICP1 and DF1 cells (*P* < 0.01, Figure 2B). These results indicate that this uORF functions as an intrinsic repressor for downstream ORF expression. To further understand the molecular mechanism underlying the repressive effect of this uORF, we quantified the relative mRNA levels of *Renilla* luciferase (*Rluc*) in cells transfected with the same amount of PPARγ1-uORF-Mut and PPARγ1-5′UTR-WT, respectively. Surprisingly, in contrast to the reporter gene assay results (Figure 2B), quantitative real-time RT-PCR showed that transfection of PPARγ1-uORF-Mut resulted in lower *Rluc* mRNA expression compared with the PPARγ1-5′UTR-WT in both ICP1 (*P* < 0.05) and DF1 cells (*P* < 0.01) (Figure 2C). Thus, the luciferase reporter gene assay and quantitative RT-PCR results together allow us to conclude that *PPAR*γ*1* uORF inhibits *Rluc* translation.

### Inhibition of PPARγ1 Translation by the uORF

**FIGURE 3:**
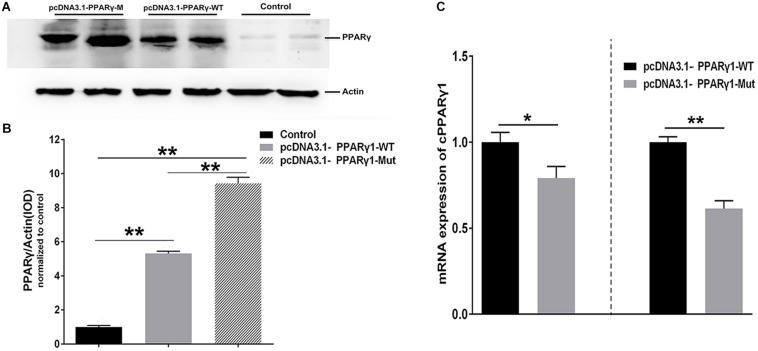
PPARγ1 translation is inhibited by its 5′ UTR uORF. **(A)** Detection of PPARγ1 protein levels. Equal amounts of the total cell lysates from the ICP1 cells transfected with either pcDNA3.1-PPARγ-WT or pcDNA3.1-PPARγ-Mut were separated and immunoblotted with an anti-PPARγ antibody. Actin was used as a loading control. **(B)** Quantification of PPARγ1 protein expression. Band intensities were measured by ImageJ software normalized to actin loading control. Data represent mean ± SEM. PPARγ1 protein expression was higher in the cells transfected with pcDNA3.1-PPARγ-Mut than in the cells transfected with the pcDNA3.1-PPARγ-WT (**P < 0.01, Student’s *t*-test). **(C)** Quantification of PPARγ1 mRNA by real-time RT-PCR in the ICP1 and DF1 cells transfected with the indicated constructs. PPARγ1 mRNA levels were normalized to the expression of the cells transfected with pcDNA3.1-PPARγ-WT. Data were expressed as the mean ± SEM, *NONO* was used as the internal mRNA control. *n* ≥ 3, *P < 0.05; **P < 0.01, Student’s *t*-test.

To exclude the possibility that the effect of this uORF is reporter gene-specific, we generated full-length PPARγ1 expression constructs with either the wild-type or uORF-mutated 5′ UTR (AUG > UAG), termed pcDNA3.1-PPARγ-WT and pcDNA3.1-PPARγ-Mut, respectively. Then, ICP1 cells were transfected with pcDNA3.1-PPARγ-WT or pcDNA3.1-PPARγ-Mut alone, and PPARγ protein expression was assayed by western blot. The western blot analysis showed that PPARγ1 protein levels were significantly higher in the ICP1 cells transfected with pcDNA3.1-PPARγ-Mut than with the pcDNA3.1-PPARγ-WT (*P* < 0.01, Figures 3A,B). In parallel, we investigated the *PPAR*γ*1* mRNA expression. Real-time RT-PCR analysis showed that *PPAR*γ*1* mRNA expression levels were significantly lower in both ICP1 and DF1 cells transfected with pcDNA3.1-PPARγ-Mut than with pcDNA3.1-PPARγ-WT (Figure 3C). These results are consistent with those of the reporter gene assay (Figure 2C). Collectively, these results indicate that this uORF represses downstream PPARγ1 translation.

### No Effect of PPARγ1 uORF on mRNA Stability

**FIGURE 4:**
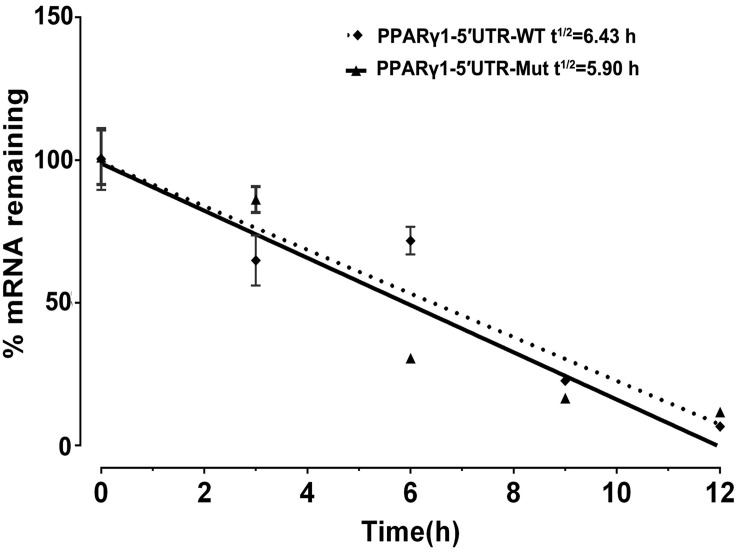
Effect of uORF mutation on Rluc mRNA stability. ICP1 cells were transiently transfected with PPARγ1-5′UTR-WT or PPARγ1-5′UTR-Mut, 48 h post-transfection, Rluc mRNA remaining after a 12 h time-course treatment with Actinomycin D was measured by real-time RT-PCR and calculated as a percentage of the level measured at time zero (0 h). Linear regression analysis was used to determine the half-life of the Rluc mRNA (*t*^1/2^), the time required for degrading 50% of the existing Rluc mRNA molecules at 0 h. No differences in relative mRNA decay rate were observed between the cells transfected with PPARγ1-5′UTR-WT and PPARγ1-5′UTR-Mut. Data were expressed as the mean ± SEM relative to NONO expression.

Our results showed that the uORF mutation resulted in reduced mRNA expression levels of *Rluc* and *PPAR*γ*1* (Figures 2C, 3C). There are two possibilities to explain this. First, the uORF mutation may affect mRNA stability. Previous studies have indicated that uORF can reduce mRNA expression via mRNA destabilization (Dikstein, 2012; Dvir et al., 2013). The other possibility is that the cloned chicken PPARγ1 5′ UTR in our 5′ UTR reporters and PPARγ expression vectors may contain promoter activity, and uORF mutation may lead to reduced promoter activity. To test whether this uORF mutation affected mRNA stability, using real-time RT-PCR, we determined the mRNA decay rate of *Rluc* in cells transfected with PPARγ1-5′UTR-WT or PPARγ1-5′UTR-Mut at 0, 3, 6, 9, and 12 h following treatment with actinomycin D. As shown in Figure 4, no significant difference in *Rluc* mRNA half-life was observed over a 12-h period between cells transfected with PPARγ1-5′UTR-WT or PPARγ1-5′UTR-Mut (PPARγ1-5′UTR-WT: 6.43 h; PPARγ1-5′UTR-Mut: 5.90 h (*P* = 0.4146). These results indicate that this uORF had no obvious effect on mRNA stability.

### The Effect of uORF Mutation on Promoter Activity

**FIGURE 5:**
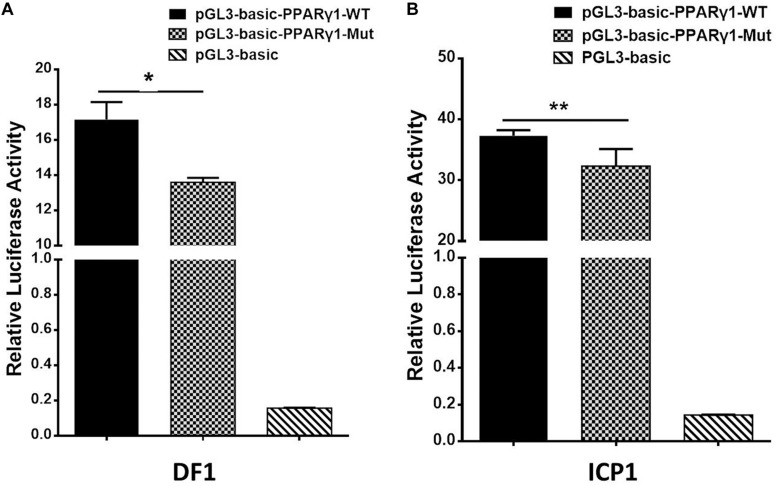
The promoter activity analysis of the DNA sequences corresponding wild-type and uORF-mutated 5′ UTRs of PPARγ1. The DNA sequences corresponding wild-type and uORF-mutated 5′ UTRs of PPARγ1 were cloned into luciferase reporter vector pGL3-basic to yield pGL3-PPARγ1-WT and pGL3-PPARγ1-Mut, respectively. The indicated reporters along with the pRL-TK *Renilla* luciferase vector were transiently transfected into DF1 **(A)** and ICP1 cells **(B)**, and the luciferase activity was determined at 48 h after transfection. The pRL-TK vector was used for normalization of transfection efficiency. All data represent the mean ± SEM. *P < 0.05, **P < 0.01, Student’s *t*-test.

The genomic region corresponding to the 5′ UTR is usually part of the promoter. Our previous study demonstrated that the 108-bp sequence downstream of the transcription start site of *PPAR*γ*1*, which is part of the 5′ UTR, had the highest promoter activity (Cui et al., 2018). To test whether the cloned PPARγ1 5′ UTR had promoter activity and whether the uORF mutation reduced it, we cloned DNA sequences corresponding to wild-type and uORF-mutated 5′ UTRs of PPARγ1 into luciferase reporter vector pGL3-Basic, named pGL3-PPARγ1-WT and pGL3-PPARγ1-Mut, respectively. A reporter gene assay showed that the pGL3-PPARγ1-WT and pGL3-PPARγ1-Mut displayed 111- and 90-fold higher luciferase reporter activity, respectively, than the pGL3-Basic empty vector in DF1 cells, and 180- and 120-fold higher luciferase reporter activity, respectively, than the pGL3-Basic empty vector in ICP1 cells, suggesting that the cloned PPARγ1 5′ UTR has promoter activity. By comparison, pGL3-PPARγ1-Mut showed significantly lower luciferase activity than pGL3-PPARγ1-WT in DF1 cells (Figure 5A, *P* < 0.05) and ICP1 cells (Figure 5B, *P* < 0.01). These results demonstrated that the cloned PPARγ1 5′ UTR had strong promoter activity and that the uORF mutation can result in reduced promoter activity. These findings explain why the mRNA expression levels of *Rluc* and *PPAR*γ*1* were reduced in the above study (Figures 2C, 3C).

### The Effect of the uORF on *in vitro* Translation of PPARγ1

**FIGURE 6:**
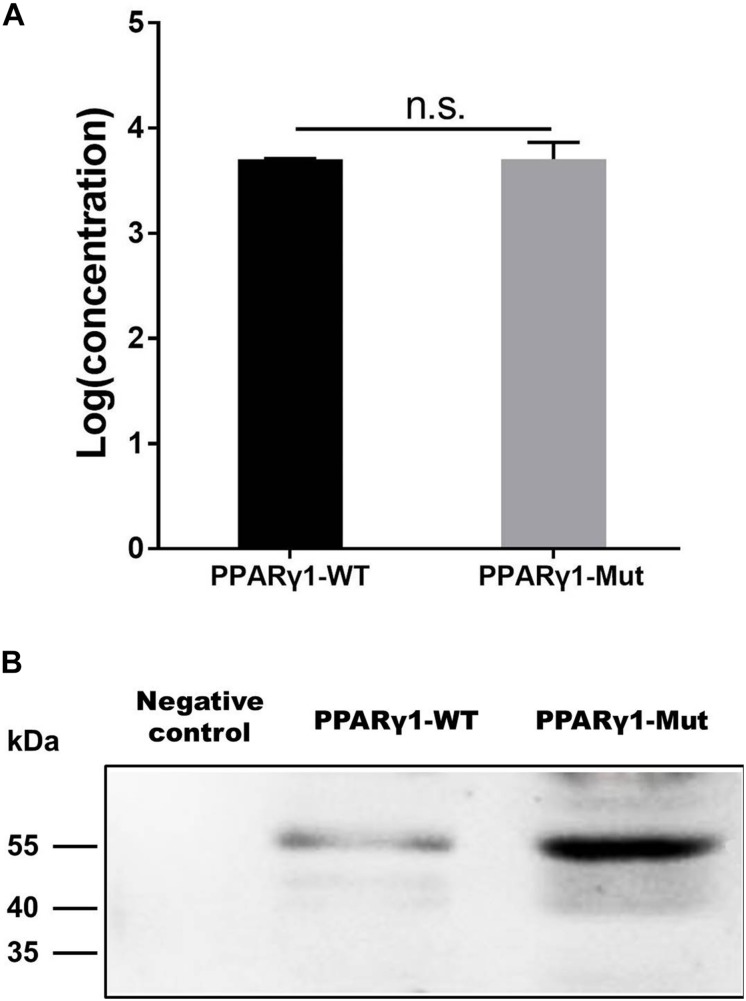
The uORF represses *in vitro* PPARγ1 translation. **(A)** *In vitro* transcribed PPARγ1 mRNAs from the wild-type and uORF-mutant PPARγ1 expression vectors (pcDNA3.1-PPARγ1-WT and pcDNA3.1-PPARγ1-Mut) were analyzed by quantitative real-time RT-PCR. No difference in PPARγ1 mRNA was observed. Data were expressed as the mean ± SEM, n.s., not significant, Student’s *t*-test. **(B)** Equal amounts of the *in vitro* transcribed mRNAs (2 μg) were used for *in vitro* translation. Note that the uORF strongly represses PPARγ1 translation. A *in vitro* translation reaction without RNA template was used as a negative control.

The results reported here suggest that at the mRNA level, the uORF represses PPARγ1 translation (Figures 3A,B). To further validate this finding, we performed an *in vitro* transcription and translation assay. For *in vitro* transcription, equal amounts (1 μg) of linearized pcDNA3.1-PPARγ1-WT or pcDNA3.1-PPARγ1-Mut were used as templates to produce the *PPAR*γ*1* mRNA with wild-type and uORF-mutated 5′ UTR using the T7 RiboMAX Large Scale RNA Production System. The results showed that, as expected, pcDNA3.1-PPARγ1-WT and pcDNA3.1-PPARγ1-Mut produced almost the same amount of PPARγ1 mRNA (Figure 6A). Equal amounts of the transcribed PPARγ1 mRNA produced from pcDNA3.1-PPARγ1-WT and pcDNA3.1-PPARγ1-Mut were used for the *in vitro* translation assay. The *in vitro* translation assay results showed that more PPARγ1 protein was synthesized with PPARγ1 mRNA from pcDNA3.1-PPARγ1-Mut than from pcDNA3.1-PPARγ1-WT (Figure 6B). Together, these results indicate that the uORF represses PPARγ1 translation.

### The PPARγ1 uORF Can Be Translated

**FIGURE 7:**
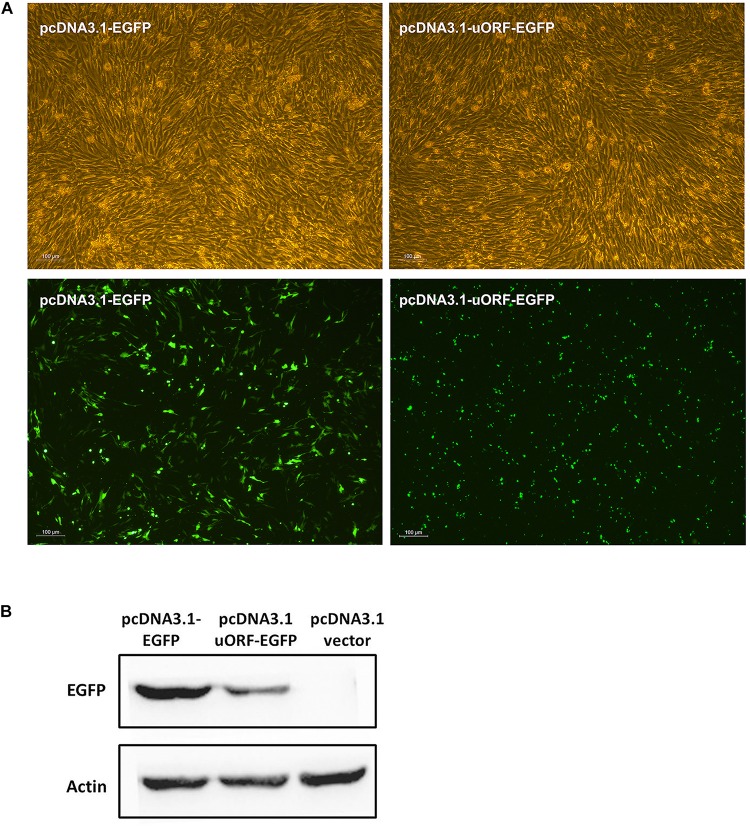
Translation can be initiated at the uAUG of the PPARγ1 uORF. **(A)** The pcDNA3.1-EGFP and pcDNA3.1-uORF-EGFP were respectively transiently transfected into ICP1 cells, 48 h post-transfection, the green fluorescence signal was visualized under a fluorescence microscope. **(B)** Lysates from the cells transfected with pcDNA3.1-EGFP and pcDNA3.1-uORF-EGFP, EGFP or uORF-EGFP fusion protein was immunoblotted with an anti-EGFP antibody. Actin was used as a loading control.

To gain insight into the molecular mechanism by which the uORF represses translation, we tested whether the uAUG of PPARγ1 uORF was used for translation initiation. We generated a construct in which the uORF was fused in frame with the enhanced green fluorescent protein (EGFP) coding sequences, with no intervening in-frame stop codons, and named it pcDNA3.1-uORF-EGFP; pcDNA3.1-EGFP was used as a positive control. The ICP1 cells were transiently transfected with pcDNA3.1-uORF-EGFP or pcDNA3.1-EGFP and examined by microscopy and western blotting. Microscopy showed that the cells transfected with either pcDNA3.1-uORF-EGFP or pcDNA3.1-EGFP displayed GFP fluorescence (Figure 7A). Comparatively, GFP fluorescence intensity was lower in the cells transfected with pcDNA3.1-uORF-EGFP than with pcDNA3.1-EGFP (Figure 7A). Consistent with these findings, western blot analysis showed that the uORF-EGFP fusion protein was expressed but at a lower level in the cells transfected with pcDNA3.1-uORF-EGFP compared with that in the cells transfected with pcDNA3.1-EGFP (Figure 7B). Collectively, these data suggest that translation can indeed be initiated at the uAUG.

## Discussion

Investigating the molecular mechanisms that control PPARγ expression is critical for understanding adipogenesis, as well as pathological conditions such as obesity and diabetes. In the present study, we investigated PPARγ posttranscriptional regulation by 5′ UTR. We demonstrated that a uORF, which is absent in human and mouse PPARγ transcripts, represses chicken PPARγ transcript variant 1 translation. To our knowledge, this is the first report of a uORF regulating *PPAR*γ gene expression.

In the present study, we demonstrated that the five chicken PPARγ 5′ UTR isoforms exerted different effects on the reporter gene activity (Figures 1A,B), and further study showed the 5′ UTR uORF of PPARγ1 represses reporter gene and PPARγ1 translation. Sequence analysis revealed that the uAUG of the PPARγ1 uORF resides in a favorable Kozak sequence context, which is the most efficient context for ribosome recognition and initiation of translation (Figure 2A). In agreement with the bioinformatics prediction, we demonstrated that the uAUG could serve as a translation start site (Figures 7A,B). Furthermore, secondary structural analysis showed there was a stable loop structure within the uORF (Supplementary Figure 1), and the uORF mutation (AUG > UAG) was not able to alter the secondary structure of PPARγ1 5′ UTR (Supplementary Figure 1). Thus, we could rule out an effect of secondary structure alteration on PPARγ1 expression.

The 17-amino acid PPARγ1 uORF peptide was analyzed using the PSIPRED protein sequence analysis workbench. It was predicted to be a disordered peptide (Romero et al., 2001; Buchan et al., 2013). Disordered peptides are enriched with residues Gly, Pro, Arg, and Ser, which are potential targets for phosphorylation that could promote ribosome stalling during translation elongation or termination (Hayden and Jorgensen, 2007; Johansson et al., 2011; Koutmou et al., 2015), which may explain why EGFP expression was lower in the cells transfected with pcDNA3.1-uORF-EGFP than with pcDNA3.1-EGFP (Figures 7A,B).

An increasing number of uORF-encoded peptides have been identified and shown to repress the downstream ORF expression by triggering ribosome stalling or suppressing reinitiation (Wilson and Beckmann, 2011; Ito and Chiba, 2013; Starck et al., 2016). Our data demonstrated that the PPARγ1 uORF repressed downstream ORF expression and that it could be translated. This raised the question of whether this uORF-encoded peptide represses downstream ORF translation. To this end, we constructed a uORF expression vector, pcDNA3.1-uORF, and co-transfected pcDNA3.1-uORF and PPARγ1-5′UTR-WT or PPARγ1-5′UTR-Mut into DF1 cells. Unexpectedly, reporter gene assays showed that transfection of pcDNA3.1-uORF increased reporter gene activities of both PPARγ1-5′UTR-WT and PPARγ1-5′UTR-Mut (Supplementary Figure 2). This result suggested that this uORF-encoded peptide may repress the downstream PPARγ1 translation in *cis*, but not in *trans.* It has been reported that several uORF-encoded peptides act in *cis* on the ribosome during their own translation to stall translation. Arrest of translation can occur either during translation elongation, as seen for SecM (Tsai et al., 2014) and VemP (Vazquez-Laslop et al., 2008), or during translation termination; for example, in the tryptophanase C (TnaC) (Gong et al., 2001) and S-adenosyl-methionine decarboxylase (SAM-DC) (Raney et al., 2002).

**FIGURE 8:**
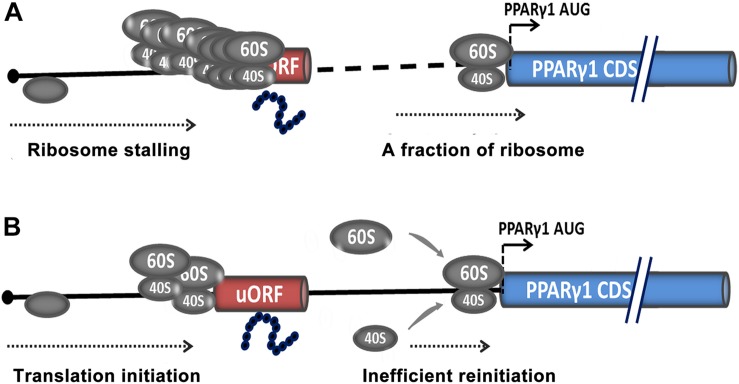
Potential models for uORF-mediated PPARγ1 translational inhibition. Translational inhibition of PPARγ1 may be due to uORF-mediated ribosome stalling **(A)** or inefficient reinitiation at the authentic start codon of PPARγ1 **(B)**.

Based on our data, we speculated that uORF repressed PPARγ1 translation by two possible mechanisms. The first was ribosome stalling (Figure 8A), in which uAUG is recognized by the scanning 40*S* ribosomal subunit and associated initiation factors, the uORF is translated, and the nascent peptide stalls the ribosome in the ribosome exit tunnel, thereby hampering the progression of upstream ribosomes (Wilson, 2011; Brandman et al., 2012; Wilson et al., 2016). Only a tiny minority of ribosomes may leaky-scan the uORF start codon and translate the PPARγ1 coding sequence. Consequently, the translational efficiency of PPARγ1 is dramatically attenuated.

The other possible mechanism is translational reinitiation, in which ribosomes translate the uORF and remain associated with the mRNA, continue scanning, and reinitiate further downstream at either a proximal or distal AUG codon (Figure 8B). However, reinitiation efficiency is substantially reduced (Roy et al., 2010; Hinnebusch et al., 2016) and translation of PPARγ1 inhibited. Recently, some nascent peptides of uORFs have been reported to be involved in the suppression of reinitiation (Ito and Chiba, 2013; Seefeldt et al., 2015). We speculate that the uORF-encoded peptide may contribute to suppression of PPARγ1 translation.

In addition, several studies have implicated that uORF-containing mRNA has the potential to trigger the nonsense-mediated decay (NMD) pathway. NMD is one of the better characterized posttranscriptional control mechanisms, whereby transcripts harboring premature translation termination codons are degraded (Mendell et al., 2004). In the present study, we detected no significant effect of uORF mutation on *Rluc* mRNA stability (Figures 4, 6A). Therefore, we can rule out the possibility that PPARγ1 uORF modulates PPARγ1 expression by triggering the NMD pathway.

PPARγ is a master regulator of adipogenesis, whole-body lipid metabolism, and insulin sensitivity. Accumulating evidence shows that adipogenesis and lipid metabolism are different between mammals and chickens (Prigge and Grande, 1971; Ji et al., 2012). For example, unlike that in mammals, chicken adipocyte lipolysis is almost exclusively regulated by glucagon, and chicken adipose tissue is not sensitive to insulin (Dupont et al., 2012; Ji et al., 2012; Wang et al., 2017). In the present study, we demonstrated that the uORF represses chicken PPARγ1 translation, and bioinformatics analysis showed that the 5′ UTR sequences of PPARγ transcripts had low sequence similarity among humans, mice, and chickens, and no uORF element was identified in the 5′ UTRs of human and mouse PPARγ transcripts. These data suggest that the posttranscriptional regulation of the *PPAR*γ gene by the 5′ UTR differs between mammals and chickens. The identified uORF element is a unique regulatory element in chicken *PPAR*γ gene, which may contribute to the differences in adipogenesis, adipose development, and insulin sensitivity between mammals and chickens. Our present study has two limitations. First, the scope of our present study is small; we just explored the regulatory role and possible mechanism of PPARγ1 uORF. Second, we did not investigate the *in vivo* importance of the PPARγ1 uORF in the regulation of PPARγ expression due to the fact that chicken gene knockout and transgenic approaches have not been well established. Given the importance of PPARγ in adipose development, obesity and related diseases, in the future we will use CRISPR-Cas9 technology to explore the *in vivo* importance and underlying molecular mechanism of PPARγ1 uORF in physiological and pathological conditions. A better understanding of PPARγ 5′ UTRs may provide clues for controlling obesity, type 2 diabetes, and insulin resistance.

In summary, for the first time, we demonstrated that a uORF represses chicken PPARγ1 translation.

## Data Availability Statement

Publicly available datasets were analyzed in this study. This data can be found here: GenBank: KP736526.1 (https://www.ncbi.nlm.nih.gov/nuccore/KP736526.1/).

## Author Contributions

YC designed the experiments, collected and analyzed the data, and wrote the manuscript. JH, GM, TC, XY, and HL participated in scientific discussions and provided technical assistances. NW supervised the study and wrote the manuscript with YC.

## Conflict of Interest

The authors declare that the research was conducted in the absence of any commercial or financial relationships that could be construed as a potential conflict of interest.

## Footnotes

1 http://star.mit.edu/index.html

2 http://itbtools.ba.itb.cnr.it/utrscan

3 http://regrna2.mbc.nctu.edu.tw

4 http://rna.tbi.univie.ac.at/cgi-bin/RNAWebSuite/RNAfold.cgi

5 http://bioinf.cs.ucl.ac.uk/psipred/

## References

[B1] Ahmadian M. Suh J. M. Hah N. Liddle C. Atkins A. R. Downes M. (2013). PPARgamma signaling and metabolism: the good, the bad and the future. *Nat. Med.* 19 557–566. 10.1038/nm.3159 23652116 PMC3870016

[B2] Aprile M. Ambrosio M. R. D’Esposito V. Beguinot F. Formisano P. Costa V. (2014). PPARG in human adipogenesis: differential contribution of canonical transcripts and dominant negative isoforms. *PPAR Res.* 2014:537865. 10.1155/2014/537865 24790595 PMC3981527

[B3] Araujo P. R. Yoon K. Ko D. Smith A. D. Qiao M. Suresh U. (2012). Before it gets started: regulating translation at the 5′ UTR. *Comp. Funct. Genomics* 2012:475731. 10.1155/2012/475731 22693426 PMC3368165

[B4] Barbosa C. Peixeiro I. Romao L. (2013). Gene expression regulation by upstream open reading frames and human disease. *PLoS Genet.* 9:e1003529. 10.1371/journal.pgen.1003529 23950723 PMC3738444

[B5] Brandman O. Stewart-Ornstein J. Wong D. Larson A. Williams C. C. Li G. W. (2012). A ribosome-bound quality control complex triggers degradation of nascent peptides and signals translation stress. *Cell* 151 1042–1054. 10.1016/j.cell.2012.10.044 23178123 PMC3534965

[B6] Buchan D. W. Minneci F. Nugent T. C. Bryson K. Jones D. T. (2013). Scalable web services for the PSIPRED protein analysis workbench. *Nucleic Acids Res.* 41 W349–W357. 10.1093/nar/gkt381 23748958 PMC3692098

[B7] Bugaut A. Balasubramanian S. (2012). 5′-UTR RNA G-quadruplexes: translation regulation and targeting. *Nucleic Acids Res.* 40 4727–4741. 10.1093/nar/gks068 22351747 PMC3367173

[B8] Calvo S. E. Pagliarini D. J. Mootha V. K. (2009). Upstream open reading frames cause widespread reduction of protein expression and are polymorphic among humans. *Proc. Natl. Acad. Sci. U.S.A.* 106 7507–7512. 10.1073/pnas.0810916106 19372376 PMC2669787

[B9] Cariou B. Charbonnel B. Staels B. (2012). Thiazolidinediones and PPARgamma agonists: time for a reassessment. *Trends Endocrinol. Metab.* 23 205–215. 10.1016/j.tem.2012.03.001 22513163

[B10] Ceraj T.J. I. Riley Shubert C. (2009). “StarHPC – Teaching parallel programming within elastic compute cloud,” in *Proceedings of the ITI 2009 31st International Conference on Information Technology Interfaces*, Cavtat.

[B11] Chandra M. Miriyala S. Panchatcharam M. (2017). PPARgamma and its role in cardiovascular diseases. *PPAR Res.* 2017:6404638. 10.1155/2017/6404638 28243251 PMC5294387

[B12] Chang T. H. Huang H. Y. Hsu B. K. Weng S. L. Horng J. T. Huang H. D. (2013). An enhanced computational platform for investigating the roles of regulatory RNA and for identifying functional RNA motifs. *BMC Bioinformatics* 14(Suppl. 2):S4–S4. 10.1186/1471-2105-14-S2-S4 23369107 PMC3549854

[B13] Chu Y. Huang J. Ma G. Cui T. Yan X. Li H. (2019). An upstream open reading frame represses translation of chicken PPARγ transcript variant 1. *bioRxiv* 10.1101/858753 PMC7058706 32184808

[B14] Crowe M. L. Wang X. Q. Rothnagel J. A. (2006). Evidence for conservation and selection of upstream open reading frames suggests probable encoding of bioactive peptides. *BMC Genomics* 7:16. 10.1186/1471-2164-7-16 16438715 PMC1402274

[B15] Cui T. Xing T. Huang J. Mu F. Jin Y. You X. (2018). Nuclear respiratory factor 1 negatively regulates the p1 promoter of the peroxisome proliferator-activated receptor-gamma gene and inhibits chicken adipogenesis. *Front. Physiol.* 9:1823. 10.3389/fphys.2018.01823 30618832 PMC6305991

[B16] Dikstein R. (2012). Transcription and translation in a package deal: the TISU paradigm. *Gene* 491 1–4. 10.1016/j.gene.2011.09.013 21983420

[B17] Duan K. Sun Y. Zhang X. Zhang T. Zhang W. Zhang J. (2015). Identification and characterization of transcript variants of chicken peroxisome proliferator-activated receptor gamma. *Poult Sci.* 94 2516–2527. 10.3382/ps/pev229 26286997

[B18] Dupont J. Metayer-Coustard S. Ji B. Rame C. Gespach C. Voy B. (2012). Characterization of major elements of insulin signaling cascade in chicken adipose tissue: apparent insulin refractoriness. *Gen. Compar. Endocrinol.* 176 86–93. 10.1016/j.ygcen.2011.12.030 22233773

[B19] Dvir S. Velten L. Sharon E. Zeevi D. Carey L. B. Weinberger A. (2013). Deciphering the rules by which 5′-UTR sequences affect protein expression in yeast. *Proc. Natl. Acad. Sci. U.S.A.* 110 E2792–E2801. 10.1073/pnas.1222534110 23832786 PMC3725075

[B20] Gong F. Ito K. Nakamura Y. Yanofsky C. (2001). The mechanism of tryptophan induction of tryptophanase operon expression: tryptophan inhibits release factor-mediated cleavage of TnaC-peptidyl-tRNA(Pro). 98 8997–9001. 10.1073/pnas.171299298 11470925 PMC55362

[B21] Grillo G. Turi A. Licciulli F. Mignone F. Liuni S. Banfi S. (2010). UTRdb and UTRsite (RELEASE 2010): a collection of sequences and regulatory motifs of the untranslated regions of eukaryotic mRNAs. *Nucleic Acids Res.* 38 D75–D80. 10.1093/nar/gkp902 19880380 PMC2808995

[B22] Hayden C. A. Jorgensen R. A. (2007). Identification of novel conserved peptide uORF homology groups in *Arabidopsis* and rice reveals ancient eukaryotic origin of select groups and preferential association with transcription factor-encoding genes. *BMC Biol.* 5:32. 10.1186/1741-7007-5-32 17663791 PMC2075485

[B23] Hinnebusch A. G. Ivanov I. P. Sonenberg N. (2016). Translational control by 5′-untranslated regions of eukaryotic mRNAs. *Science* 352 1413–1416. 10.1126/science.aad9868 27313038 PMC7422601

[B24] Hofacker I. L. (2003). Vienna RNA secondary structure server. *Nucleic Acids Res.* 31 3429–3431. 10.1093/nar/gkg599 12824340 PMC169005

[B25] Hood H. M. Neafsey D. E. Galagan J. Sachs M. S. (2009). Evolutionary roles of upstream open reading frames in mediating gene regulation in fungi. *Annu. Rev. Microbiol.* 63 385–409. 10.1146/annurev.micro.62.081307.162835 19514854

[B26] Iacono M. Mignone F. Pesole G. (2005). uAUG and uORFs in human and rodent 5′untranslated mRNAs. *Gene* 349 97–105. 10.1016/j.gene.2004.11.041 15777708

[B27] Ito K. Chiba S. (2013). Arrest peptides: cis-acting modulators of translation. *Annu. Rev. Biochem.* 82 171–202. 10.1146/annurev-biochem-080211-105026 23746254

[B28] Ji B. Ernest B. Gooding J. R. Das S. Saxton A. M. Simon J. (2012). Transcriptomic and metabolomic profiling of chicken adipose tissue in response to insulin neutralization and fasting. *BMC Genomics* 13:441. 10.1186/1471-2164-13-441 22938590 PMC3503602

[B29] Johansson M. Ieong K. W. Trobro S. Strazewski P. Aqvist J. Pavlov M. Y. (2011). pH-sensitivity of the ribosomal peptidyl transfer reaction dependent on the identity of the A-site aminoacyl-tRNA. *Proc. Natl. Acad. Sci. U.S.A.* 108 79–84. 10.1073/pnas.1012612107 21169502 PMC3017169

[B30] Katsura S. Okumura T. Ito R. Sugawara A. Yokoyama A. (2014). Identification of posttranslational modifications in peroxisome proliferator-activated receptor gamma using mass spectrometry. *PPAR Res.* 2014:468925. 10.1155/2014/468925 25061449 PMC4099357

[B31] Koutmou K. S. Schuller A. P. Brunelle J. L. Radhakrishnan A. Djuranovic S. Green R. (2015). Ribosomes slide on lysine-encoding homopolymeric A stretches. *Elife* 4:e05534. 10.7554/eLife.05534 25695637 PMC4363877

[B32] Lee J. E. Ge K. (2014). Transcriptional and epigenetic regulation of PPARgamma expression during adipogenesis. *Cell Biosci.* 4:29. 10.1186/2045-3701-4-29 24904744 PMC4046494

[B33] Lefterova M. I. Haakonsson A. K. Lazar M. A. Mandrup S. (2014). PPARgamma and the global map of adipogenesis and beyond. *Trends Endocrinol. Metab.* 25 293–302. 10.1016/j.tem.2014.04.001 24793638 PMC4104504

[B34] Lehrke M. Lazar M. A. (2005). The many faces of PPARgamma. *Cell* 123 993–999. 10.1016/j.cell.2005.11.026 16360030

[B35] Livak K. J. Schmittgen T. D. (2001). Analysis of relative gene expression data using real-time quantitative PCR and the 2(-Delta Delta C(T)) method. *Methods* 25 402–408. 10.1006/meth.2001.1262 11846609

[B36] Mcclelland S. Shrivastava R. Medh J. D. (2014). Regulation of translational efficiency by disparate 5′ UTRs of PPARgamma splice variants. *Ppar Res.* 2009:193413. 10.1155/2009/193413 20016806 PMC2792960

[B37] Mendell J. T. Sharifi N. A. Meyers J. L. Martinez-Murillo F. Dietz H. C. (2004). Nonsense surveillance regulates expression of diverse classes of mammalian transcripts and mutes genomic noise. *Nat. Genet.* 36 1073–1078. 10.1038/ng1429 15448691

[B38] Mitchell S. F. Walker S. E. Algire M. A. Park E. H. Hinnebusch A. G. Lorsch J. R. (2010). The 5′-7-methylguanosine cap on eukaryotic mRNAs serves both to stimulate canonical translation initiation and to block an alternative pathway. *Mol. Cell.* 39 950–962. 10.1016/j.molcel.2010.08.021 20864040 PMC2945613

[B39] Nasif S. Contu L. Muhlemann O. (2018). Beyond quality control: the role of nonsense-mediated mRNA decay (n.d.) in regulating gene expression. *Semin. Cell Dev Biol.* 75 78–87. 10.1016/j.semcdb.2017.08.053 28866327

[B40] Normann K. R. Øystese K. A. B. Berg J. P. Lekva T. Berg-Johnsen J. Bollerslev J. (2016). Selection and validation of reliable reference genes for RT-qPCR analysis in a large cohort of pituitary adenomas. *Mol. Cell. Endocrinol.* 437(C) 183–189. 10.1016/j.mce.2016.08.030 27561203

[B41] Prigge W. F. Grande F. (1971). Effects of glucagon, epinephrine and insulin on in vitro lipolysis of adipose tissue from mammals and birds. *Compar. Biochem. Physiol. B* 39 69–82. 10.1016/0305-0491(71)90254-9 5570026

[B42] Raney A. Law G. L. Mize G. J. Morris D. R. (2002). Regulated translation termination at the upstream open reading frame in s-adenosylmethionine decarboxylase mRNA. *J. Biol. Chem.* 277 5988–5994. 10.1074/jbc.M108375200 11741992

[B43] Romero P. Obradovic Z. Li X. Garner E. C. Brown C. J. Dunker A. K. (2001). Sequence complexity of disordered protein. *Proteins* 42 38–48. 11093259 10.1002/1097-0134(20010101)42:1<38::aid-prot50>3.0.co;2-3

[B44] Roy B. Vaughn J. N. Kim B. H. Zhou F. Gilchrist M. A. Von Arnim A. G. (2010). The h subunit of eIF3 promotes reinitiation competence during translation of mRNAs harboring upstream open reading frames. *RNA* 16 748–761. 10.1261/rna.2056010 20179149 PMC2844622

[B45] Sathirapongsasuti J. F. Sathira N. Suzuki Y. Huttenhower C. Sugano S. (2011). Ultraconserved cDNA segments in the human transcriptome exhibit resistance to folding and implicate function in translation and alternative splicing. *Nucleic Acids Res.* 39 1967–1979. 10.1093/nar/gkq949 21062826 PMC3064809

[B46] Seefeldt A. C. Nguyen F. Antunes S. Perebaskine N. Graf M. Arenz S. (2015). The proline-rich antimicrobial peptide Onc112 inhibits translation by blocking and destabilizing the initiation complex. *Nat. Struct. Mol. Biol.* 22 470–475. 10.1038/nsmb.3034 25984971

[B47] Starck S. R. Tsai J. C. Chen K. Shodiya M. Wang L. Yahiro K. (2016). Translation from the 5′ untranslated region shapes the integrated stress response. *Science* 351:aad3867. 10.1126/science.aad3867 26823435 PMC4882168

[B48] Suzuki Y. Ishihara D. Sasaki M. Nakagawa H. Hata H. Tsunoda T. (2000). Statistical analysis of the 5′ untranslated region of human mRNA using “oligo-capped” cDNA libraries. *Genomics* 64 286–297. 10.1006/geno.2000.6076 10756096

[B49] Tsai A. Kornberg G. Johansson M. Chen J. Puglisi J. (2014). The dynamics of secm-induced translational stalling. *Cell Rep.* 7 1521–1533. 10.1016/j.celrep.2014.04.033 24836001 PMC4059775

[B50] van Beekum O. Fleskens V. Kalkhoven E. (2009). Posttranslational modifications of PPAR-gamma: fine-tuning the metabolic master regulator. *Obesity (Silver Spring)* 17 213–219. 10.1038/oby.2008.473 19169221

[B51] Vazquez-Laslop N. Thum C. Mankin A. S. (2008). Molecular mechanism of drug-dependent ribosome stalling. *Mol. Cell* 30 190–202. 10.1016/j.molcel.2008.02.026 18439898

[B52] Wang W. Zhang T. Wu C. Wang S. Wang Y. Li H. (2017). Immortalization of chicken preadipocytes by retroviral transduction of chicken TERT and TR. *PLoS ONE* 12:e0177348. 10.1371/journal.pone.0177348 28486516 PMC5423695

[B53] Wen Y. Liu Y. Xu Y. Zhao Y. Hua R. Wang K. (2009). Loss-of-function mutations of an inhibitory upstream ORF in the human hairless transcript cause Marie Unna hereditary hypotrichosis. *Nat. Genet.* 41 228–233. 10.1038/ng.276 19122663

[B54] Wilson D. N. (2011). Peptides in the ribosomal tunnel talk back. *Mol. Cell* 41 247–248. 10.1016/j.molcel.2011.01.017 21292157

[B55] Wilson D. N. Arenz S. Beckmann R. (2016). Translation regulation via nascent polypeptide-mediated ribosome stalling. *Curr. Opin. Struct. Biol.* 37 123–133. 10.1016/j.sbi.2016.01.008 26859868

[B56] Wilson D. N. Beckmann R. (2011). The ribosomal tunnel as a functional environment for nascent polypeptide folding and translational stalling. *Curr. Opin. Struct. Biol.* 21 274–282. 10.1016/j.sbi.2011.01.007 21316217

[B57] Xia X. Holcik M. (2009). Strong eukaryotic IRESs have weak secondary structure. *PLoS ONE* 4:e4136. 10.1371/journal.pone.0004136 19125192 PMC2607549

[B58] Yamashita R. Suzuki Y. Takeuchi N. Wakaguri H. Ueda T. Sugano S. (2008). Comprehensive detection of human terminal oligo-pyrimidine (TOP) genes and analysis of their characteristics. *Nucleic Acids Res.* 36 3707–3715. 10.1093/nar/gkn248 18480124 PMC2441802

