# Supplementary material for: An Upstream Open Reading Frame Represses Translation of Chicken PPARγ Transcript Variant 1
Source: Front Genet. 2020 Feb 28;11:165. doi: 10.3389/fgene.2020.00165 (PMC7058706; doi:10.3389/fgene.2020.00165)
Supplement: Supplementary file 1 [file Data_Sheet_1.PDF]

Supplementary Figures for

An upstream open reading frame represses translation of chicken PPARγ transcript variant 1

Yankai Chu[1,2,3], Jiaxin Huang[1,2,3], Guangwei Ma[1,2,3], Tingting Cui[1,2,3], Xiaohong Yan[1,2,3], Hui Li[1,2,3], Ning Wang[1,2,3]*

[1] Key Laboratory of Chicken Genetics and Breeding, Ministry of Agriculture and Rural Affairs, Harbin, China

[2] Key Laboratory of Animal Genetics, Breeding and Reproduction, Education Department of Heilongjiang Province, Harbin, China

[3]College of Animal Science and Technology, Northeast Agricultural University, Harbin, China

Running title: An upstream ORF represses chicken PPARγ1 translation

***Correspondence:**

Ning Wang (email: wangning@neau.edu.cn)

Keywords: PPARγ, 5′ untranslated region (5′ UTR), upstream open reading frame (uORF), translational repression, gene expression

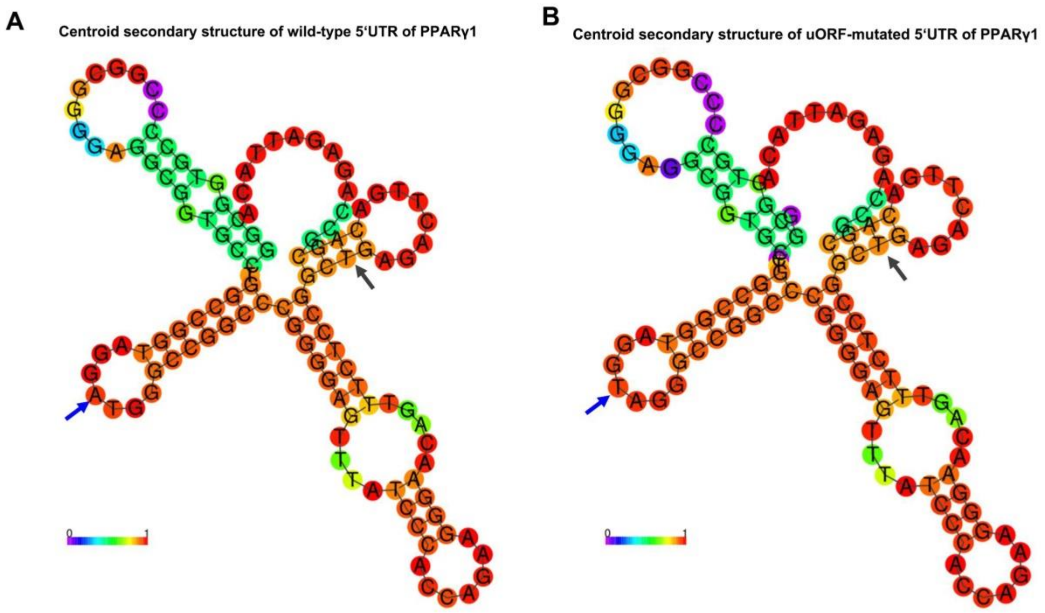

Supplementary Figure 1. Predicted secondary structure of the wild-type and uORF-mutant 5′ UTR of chicken *PPARγ1* mRNA.

RNAfold 2.0 (http://rna.tbi.univie.ac.at/cgi-bin/RNAfold.cgi) was used for structure prediction. The centroid structures encoding base pair probabilities are shown. The bases are colored in violet (0) for low and in *red* (1) for high base-pairing probabilities. The uORF within the 5′UTR are indicated by blue (5′ terminal) and black (3′ terminal) *arrows*.

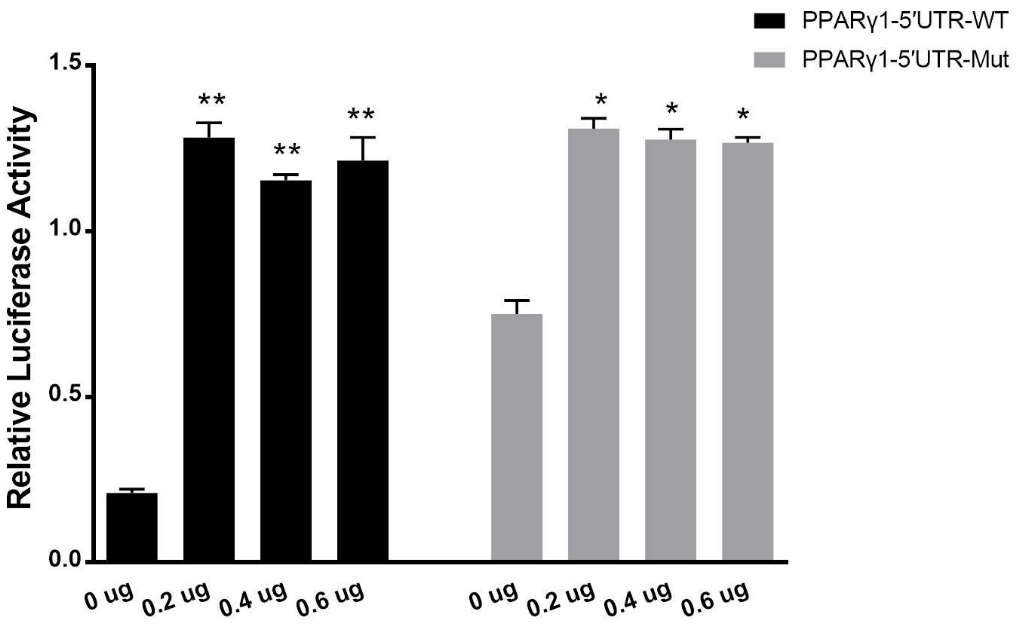

Supplementary Figure 2. The uORF-encoded peptide does not repress the downstream reporter gene translation in *trans*.

The indicated amounts of the uORF expression vector (pcDNA3.1-uORF) and either PPARγ1-5′UTR-WT or PPARγ1-5′UTR-Mut were contransfected into DF1 cells, respectively, and the luciferase activity was determined at 48 h after transfection. All data represent the mean ± SEM. *$p < 0.05$, Student's *t*-test.
